# *Cyfip1* Haploinsufficiency Increases Compulsive-Like Behavior and Modulates Palatable Food Intake in Mice: Dependence on *Cyfip2* Genetic Background, Parent-of Origin, and Sex

**DOI:** 10.1534/g3.119.400470

**Published:** 2019-07-19

**Authors:** Richard K. Babbs, Jacob A. Beierle, Qiu T. Ruan, Julia C. Kelliher, Melanie M. Chen, Ashley X. Feng, Stacey L. Kirkpatrick, Fabiola A. Benitez, Fred A. Rodriguez, Johanne J. Pierre, Jeya Anandakumar, Vivek Kumar, Megan K. Mulligan, Camron D. Bryant

**Affiliations:** * Laboratory of Addiction Genetics, Department of Pharmacology and Experimental Therapeutics and Psychiatry; † T32 NIGMS Training Program in Biomolecular Pharmacology; ‡ Boston University’s Transformative Training Program in Addiction Science (TTPAS), Biomedical Genetics, Boston University School of Medicine, Boston, MA 02118; § The Jackson Laboratory, 600 Main St., Bar Harbor, ME 04609, and; ** Department of Genetics, Genomics, and Informatics, University of Tennessee Health Science Center, 71 S. Manassas St, Memphis, TN 38163

**Keywords:** C57BL/6 substrains, binge eating disorder, overeating, anorexia nervosa, Fragile X, FMRP, psychiatric genetics, addiction genetics, neuropsychiatric, Genetics of Sex

## Abstract

Binge eating (BE) is a heritable trait associated with eating disorders and involves episodes of rapid, large amounts of food consumption. We previously identified cytoplasmic FMR1-interacting protein 2 (*Cyfip2*) as a genetic factor underlying compulsive-like BE in mice. *CYFIP2* is a homolog of *CYFIP1* which is one of four paternally-deleted genes in patients with Type I Prader-Willi Syndrome (PWS), a neurodevelopmental disorder whereby 70% of cases involve paternal 15q11-q13 deletion. PWS symptoms include hyperphagia, obesity (if untreated), cognitive deficits, and obsessive-compulsive behaviors. We tested whether *Cyfip1* haploinsufficiency (+/−) would enhance compulsive-like behavior and palatable food (PF) intake in a parental origin- and sex-dependent manner on two *Cyfip2* genetic backgrounds, including the BE-prone C57BL/6N (*Cyfip2*^N/N^) background and the BE-resistant C57BL/6J (*Cyfip2*^J/J^) background. *Cyfip1*^+/−^ mice showed increased compulsive-like behavior on both backgrounds and increased PF intake on the *Cyfip2*^N/N^ background. In contrast, maternal *Cyfip1* haploinsufficiency on the BE-resistant *Cyfip2*^J/J^ background induced a robust escalation in PF intake in wild-type *Cyfip1*^J/J^ males while having no effect in *Cyfip1*^J/-^ males. Notably, induction of behavioral phenotypes in wild-type males following maternal *Fmr1*^+/−^ has previously been reported. In the hypothalamus, there was a paternally-enhanced reduction in CYFIP1 protein whereas in the nucleus accumbens, there was a maternally-enhanced reduction in CYFIP1 protein. Nochange in FMR1 protein (FMRP) was observed in *Cyfip1*^+/−^ mice, regardless of parental origin. To summarize, *Cyfip1* haploinsufficiency increased compulsive-like behavior and induced genetic background-dependent, sex-dependent, and parent-of-origin-dependent effects on PF consumption and CYFIP1 expression that could have relevance for neurodevelopmental and neuropsychiatric disorders.

Binge eating (**BE**) refers to the rapid consumption of large quantities of food and is accompanied by feelings of loss of control. Binge eating disorder (**BED**) is a psychiatric disorder with a lifetime prevalence of 3.5% in women and 2% in men (Hudson *et al.* 2007). Both BED (Mitchell *et al.* 2010) and BE are heritable (Bulik *et al.* 2003). However, genome-wide association studies have yet to identify genetic risk factors associated with BE (Yilmaz *et al.* 2015). The first genome-wide significant loci were recently identified for anorexia nervosa (restricted eating) (Hinney *et al.* 2017) and bipolar disorder with BE behavior (PRR5-ARHGAP8) (McElroy *et al.* 2018). Additional genome-wide significant loci will likely soon be uncovered for BE-associated disorders with increasing sample sizes and power (Huckins *et al.* 2018).

We used quantitative trait locus (**QTL**) mapping and gene knockout in C57BL/6 mouse substrains to identify cytoplasmic FMR1-interacting protein 2 (*Cyfip2*) as a major genetic factor underlying BE and compulsive-like behaviors (Kirkpatrick *et al.* 2017). The QTL capturing increased palatable food (PF) intake mapped to a single missense mutation in *Cyfip2* in the C57BL/6N strain (S968F; “*Cyfip2*^M1N^”) that is hypothesized to act as a gain-of-function mutation (Kumar *et al.* 2013). Accordingly, mice with one copy of a null allele and one copy of the missense allele of *Cyfip2* showed a reduction in BE toward the phenotypic direction of the wild-type C57BL/6J level (Kirkpatrick *et al.* 2017). This same missense SNP in *Cyfip2* was first associated with reduced behavioral sensitivity to cocaine (Kumar *et al.* 2013), which could indicate a common neurobiological mechanism involving synaptic plasticity within the mesocorticolimbic dopamine reward pathway (Bello and Hajnal. 2010; Berridge. 2009) that affects the hedonic component of PF consumption (DiLeone *et al.* 2012; Lutter and Nestler 2009).

*Cyfip2* and the gene homolog *Cyfip1* code for proteins that interact with the RNA binding protein Fragile X Mental Retardation Protein (**FMRP**) and are part of the canonical WAVE regulatory complex and transduce activity-dependent Rac signaling in regulating actin dynamics during neuronal development and synaptic plasticity (Abekhoukh and Bardoni. 2014). CYFIP1 expression is necessary for the maintenance and stabilization of neuronal dendritic arborization and morphological complexity (Pathania *et al.* 2014). In humans, *CYFIP1* resides within a non-imprinted region on chromosome 15 (15q11.2) that contains four genes *TUBGCP5*, *NIPA1*, *NIPA2*, and *CYFIP1* (Bittel *et al.* 2006). The syntenic region in mice is located on chromosome 7C (55.4 Mb - 56 Mb). Preclinical models of *Cyfip1* haploinsufficiency demonstrate perturbations in synaptic activity during neural development, activity-dependent plasticity, dendritic morphology, and fear learning (Bozdagi *et al.* 2012; Chung *et al.* 2015; Hsiao *et al.* 2016; Oguro-Ando *et al.* 2015). Haploinsufficiency of 15q11.2 underlies Microdeletion Syndrome (**MDS**; a.k.a. Burnside-Butler Syndrome) which can comprise developmental delay (speech, motor), reduced cognitive function, dysmorphic features, intellectual disability, autism, ADHD, obsessive-compulsive disorder, and schizophrenia (Cox and Butler. 2015). One case study of 15q11.2 MDS reported hypotonia, increased food craving and obesity, and obsessive-compulsive disorder (Doornbos *et al.* 2009). *CYFIP1* haploinsufficiency is implicated in multiple symptoms of 15q11.2 MDS and a new study demonstrates parent-of origin effects the microdeletion on the distribution of clinical features (Davis *et al.* 2019). Converse to microdeletion, microduplication of 15q11.2 containing *CYFIP1* was recently associated anorexia nervosa (Chang *et al.* 2019).

The 15q11.2 region is also paternally-deleted in a subset of individuals with a more severe form (Type I) of Prader-Willi Syndrome (**PWS**), a neurodevelopmental disorder defined genetically by paternal deletion of 15q11-q13 in a majority of cases (Angulo *et al.* 2015). Extreme hyperphagia due to lack of satiety is the most defining and debilitating feature of PWS and emerges during childhood, leading to obesity if left untreated. Food-related obsessive-compulsive (**OC**) behaviors are common in PWS; however, OC symptoms unrelated to food are also frequent (State *et al.* 1999), and include repetitive, ritualistic behaviors, perseverative speech, counting, adaptive impairment, need to tell, ask, or know, ordering and arranging, repeating rituals, and self-mutilation (Dykens *et al.* 1996; Feurer *et al.* 1998; Stein *et al.* 1994). Genetic deletion in PWS involves either the shorter paternal deletion (Type II) of 15q11-q13 or a larger, paternal Type I deletion that also includes the same 15q11.2 MDS region comprising *TUBGCP5*, *NIPA1*, *NIPA2*, and *CYFIP1* (Bittel *et al.* 2006; Butler *et al.* 2004). Type I PWS is associated with reduced transcription of these genes and a more severe neurodevelopmental and neuropsychiatric profile, including reduced cognition, increased risk of autism and schizophrenia, and increased severity and lack of control over OC behaviors (*e.g.*, grooming and bathing, arranging objects, object hoarding, checking) that interfere with social functioning (Bittel *et al.* 2006; Butler *et al.* 2004; Doornbos *et al.* 2009; Milner *et al.* 2005; Zarcone *et al.* 2007).

Decreased CYFIP1 expression is also implicated in the Prader-Willi Phenotype (**PWP**) of a subset of individuals with Fragile-X Syndrome (**FXS**). FXS is the most common genetic cause of intellectual disability and autism and is caused by a CGG trinucleotide repeat expansion within the fragile X mental retardation 1 (*FMR1*) gene that is located on the X chromosome and codes for FMRP, a major interacting protein of CYFIP proteins (Schenck *et al.* 2001). Interestingly, 10% of FXS individuals also exhibit a PWP in the absence structural or imprinting differences in 15q11-q13. The PWP includes hallmark hyperphagia, lack of satiation, obesity, and more severe behavioral problems, such as OC behaviors and an increased rate of autism (Muzar *et al.* 2016; Nowicki *et al.* 2007). The cause of the PWP is unknown, although one logical candidate gene is *CYFIP1*, given its association with PWS and its interaction with FMRP (Schenck *et al.* 2001). PWP-presenting individuals with FXS show a two-to fourfold decrease in CYFIP1 transcription in peripheral blood mononuclear cells compared to FXS individuals without PWP (Nowicki *et al.* 2007). There was also a twofold decrease in *Cyfip1* gene transcription in a mouse model of FXS (Stefan *et al.* 2005).

We previously mapped the gene homolog *Cyfip2* in BE (Kirkpatrick *et al.* 2017). Because *CYFIP1* deletion and reduced CYFIP1 expression are associated with more severe PWS (Type I) and hyperphagia in the PWP (FXS) and conversely, because gene duplication and thus, increased expression of *CYFIP1* are associated with restrictive eating (Chang *et al.* 2019), in this study, we tested the hypothesis that *Cyfip1* haploinsufficiency would increase premorbid compulsive-like behavior and increase consumption of palatable food (**PF**) in our BE paradigm (Babbs *et al.* 2018; Goldberg *et al.* 2017; Kirkpatrick *et al.* 2017). We tested the effect of *Cyfip1* haploinsufficiency on two different *Cyfip2* genetic backgrounds. Additionally, because a recent preclinical study demonstrated a parental origin (**PO**) effect of *Cyfip1* haploinsufficiency on hippocampal synaptic transmission, learning, and anxiety-like behavior (Chung *et al.* 2015) and because a recent clinical study indicated an effect of PO on the distribution of clinical features in 15q11.2 MDS (Burnside-Butler Syndrome) (Davis *et al.* 2019), we tested whether there would be an effect of PO of *Cyfip1* deletion on compulsive-like behavior and PF intake.

To gain insight into the molecular mechanisms associated with PO effects of *Cyfip1* deletion on PF intake, we examined transcription of *Cyfip1*, *Cyfip2*, and *Magel2* - a nearby imprinted gene within the syntenic, canonical PWS region that is implicated in hyperphagia and obesity (Tacer and Potts. 2017). Additionally, we examined protein expression of CYFIP1 and its interacting partner FMRP as a function of both *Cyfip1* genotype and PO in two brain regions, including the hypothalamus which is critical for homeostatic regulation of feeding and the nucleus accumbens which is critical for hedonic aspects of food intake (DiLeone *et al.* 2012; Lutter and Nestler. 2009). Finally, because OC behaviors are associated with BE (Kessler *et al.* 2016; Moore *et al.* 2017; Wilfley *et al.* 2016) and hyperphagia in PWS (Griggs *et al.* 2015), we employed a battery of tests to assess anxiety-like and compulsive-like behaviors and post-BE training behaviors in *Cyfip1* haploinsufficient mice, including compulsive-like eating and concomitant behaviors in the light/dark conflict test (Babbs *et al.* 2018; Kirkpatrick *et al.* 2017).

## Materials And Methods

### Mice

All experiments were performed in accordance with the National Institutes of Health Guidelines for the Use of Laboratory Animals and were approved by the Institutional Animal Care and Use Committee at Boston University. Mice were 50-100 days old at the first day of testing. A minimum sample size of N = 20 per Genotype per Treatment was employed for behavioral studies based on power analysis of PF intake from the *Cyfip2* study (Kirkpatrick *et al.* 2017) (see Supplementary Material for additional details on power analyses). Mice heterozygous for a null deletion in exons 4 through 6 of *Cyfip1* (*Cyfip1*^+/−^) were generated by the International Knockout Mouse Consortium on C57BL/6N background (Skarnes *et al.* 2011). We propagated these mice on two different C57BL/6 genetic backgrounds, including the BE-prone isogenic C57BL6/N background or on a mixed background C57BL/6J / C57BL/6N background whereby mice were backcrossed to C57BL/6J to be homozygous for the BE-resistant C57BL6/J allele at the *Cyfip2* locus. Additional details regarding mouse breeding and genotyping of *Cyfip1* and *Cyfip2* are provided in the Supplementary Material.

### Premorbid anxiety-like and compulsive-like behavioral battery

Because of the link between anxiety, compulsivity and pathological overeating (Moore *et al.* 2017) and because OC behavior is associated with eating disorders (Cavallini *et al.* 2000; Micali *et al.* 2011), we incorporated a behavioral battery to assess differences in premorbid anxiety-like and compulsive-like behaviors in experimentally naïve, *Cyfip1*^+/−^ mice. Mice were tested in the behavioral battery and were either killed afterward (all mice on *Cyfip1,2*^N/N^ background) or were subsequently trained for BE (a subset of mice on the *Cyfip1,2*^J/J^ background). Mice were assayed in the battery with one test per day over five days in the following order: 1) open field; 2) elevated plus maze; 3) marble burying; 4) hole board; 5) mist-induced grooming. The rationale and procedural details for each behavioral test are provided in the Supplementary Material. Testing was conducted between 0800 and 1300 h. The experimenters responsible for running the mice, video tracking, data curation, and analysis were blinded to Genotype for each cohort.

### BE and light/dark conflict test of compulsive-like eating

Mice were trained in an intermittent, limited access, conditioned place preference (**CPP**) procedure to detect genetic differences in BE (Babbs *et al.* 2018; Goldberg *et al.* 2017; Kirkpatrick *et al.* 2017). All mice on the *Cyfip1*,2^N/N^ background were experimentally naïve prior to BE training. For mice on the *Cyfip1*,2^J/J^ background, approximately one-half of the total sample size had previously undergone testing in the five-day behavioral battery (described above) prior to commencement of BE training. Effects of prior battery testing on food intake are described below in the Results section.

Initial locomotor activity was also quantified on Day (D)1 prior to BE training. For details on the BE protocol, see Supplementary Material. Briefly, mice were tested for side preference on D1 and D22. During the intervening three weeks, mice were confined to a food-paired and non-food-paired side on alternating days (Tuesday through Friday). Cages were assigned to either the PF or Chow group in a counterbalanced design in order to ensure equal distribution across Sex, Genotype, Treatment, and PO. On D23, mice were assessed for compulsive eating and associated behaviors, as previously described (Babbs *et al.* 2018; Kirkpatrick *et al.* 2017). (Supplementary Material). The experimenters responsible for running the mice, video tracking, data curation, and analysis were blinded to Genotype for each cohort.

### Hypothalamus dissections for real-time quantitative PCR (qPCR)

We chose a subset of Chow-trained, PF-naive mice (n = 7-9 per Genotype per PO; both sexes) on the *Cyfip1,2*^N/N^ background or untrained, naïve mice on a *Cyfip1,2*^J/J^ background (n = 8-12 per Genotype per PO; both sexes) to examine baseline (PF-naive) gene transcription between *Cyfip1*^N/-^ *vs. Cyfip1*^N/N^ mice and PO effects. We examined *Cyfip1*, *Cyfip2*, and *Magel2* transcript levels in the hypothalamus, a brain region important for hyperphagia in PWS (Griggs *et al.* 2015).

On D24, brains from Chow-trained mice (*Cyfip1,2*^N/N^ background) were harvested and the hypothalamus was free form dissected by pinching the entire structure from the ventral surface with forceps while using the anterior commissure and mammillary bodies as landmarks. Tissue was stored in RNAlater Solution (Invitrogen, Carlsbad, CA USA) at 4°. After five days, the tissue was dried and transferred to a -80° freezer.

### Real-time quantitative PCR (qPCR)

Total RNA from hypothalamus was extracted and processed for qPCR as described (Goldberg *et al.* 2017; Kirkpatrick *et al.* 2017; Yazdani *et al.* 2015). Briefly, oligo-dT primers were used to synthesize cDNA. PCR reactions were conducted on the StepOne Plus 96-Well Real-Time PCR machine (Life Technologies, Foster City, CA, USA) in technical triplicates and averaged (SD < 0.5). Plates were balanced across Genotype, PO, and Sex. We report the difference in expression in *Cyfip1*^+/−^ relative to *Cyfip1*^+/+^ using the 2^-(∆∆CT)^ method (Schmittgen and Livak. 2008). Primer sequences are provided in the Supplementary Material. All qPCR samples analyzed on the *Cyfip1,2*^N/N^ background were from Chow-trained mice. All qPCR samples analyzed on the *Cyfip1,2^J^*^/J^ genetic background were from experimentally naïve mice.

### Immunoblot analysis of CYFIP1 and FMRP From hypothalamus and nucleus accumbens

Hypothalamus was dissected as described above. Nucleus accumbens punches were harvested using 1.2 mm-diameter punches of ventral forebrain centered around anterior commissure from the first 4 mm of brain section in a brain matrix. Samples were processed and analyzed for quantity of CYFIP1 and FMRP proteins. A majority of the tissue samples for immunoblot analysis were collected from PF-trained mice. In addition to the collapsed analysis across PF and Chow samples that we report below, we conducted a separate analysis that excluded the Chow-trained samples and obtained qualitatively the same trending results for CYFIP1 and the same null results for FMRP (also described below). Thus, the addition of Chow samples improved our statistical power without confounding the results. Detailed methods can be found in the Supplementary Material.

### Analysis

Statistical analyses were conducted using R (https://www.r.project.org). For the compulsive-like and anxiety-like behavioral tests, two-tailed unpaired *t*-tests were used to detect genotypic differences for all behaviors except marble burying behaviors which were also analyzed by non-parametric Mann-Whitney *U*-tests.

Food intake was analyzed using various mixed model ANOVAs that included Genotype, Treatment, Sex, and/or PO as independent variables as indicated in each section below, and Day as a repeated measure using the “aov” function in R. Subsequent follow-up ANOVAs and *t*-tests were run to determine the source of various interactions among these variables as indicated below. To address issues of non-normality or unequal variance, we included additional, select non-parametric analyses to support key findings that support our conclusions (Supplementary Material).

Slope analyses of food intake across days were calculated to detect escalation in consumption as a measure of BE-like behavior (Babbs *et al.* 2012; Babbs *et al.* 2018; Kirkpatrick *et al.* 2017) using GraphPad Prism 7 (GraphPad Software, La Jolla, CA USA). Linear regression was employed to fit a line for each group, to calculate the slope and y-intercept, and to determine whether each slope was significantly different from zero. Group differences in slopes were detected using Analysis of Covariance (ANCOVA) and *post hoc* pairwise, Bonferroni-adjusted comparisons. Slope of escalation in food intake was included as an additional analysis in order to represent the degree of escalation of food consumption over time. A positive (non-zero) slope indicates significant escalation, whereas no slope indicates no escalation. The y-intercept is affected by both initial consumption and the degree of stability in average consumption over time. For example, a slope that was not significantly different from zero could be explained by greater initial consumption for the early training trials that was maintained at a similar over time. Significant differences in y-intercepts were cross-referenced with the daily intake values to interpret the results.

### Data Availability

The authors affirm that all data necessary for confirming the conclusions of this article are represented fully within the article and its tables and figures. Supplemental material available at Figshare: https://doi.org/10.25387/g3.8316635.

## Results

### Cyfip1 haploinsufficiency increases compulsive-like behaviors

**Figure 1:**
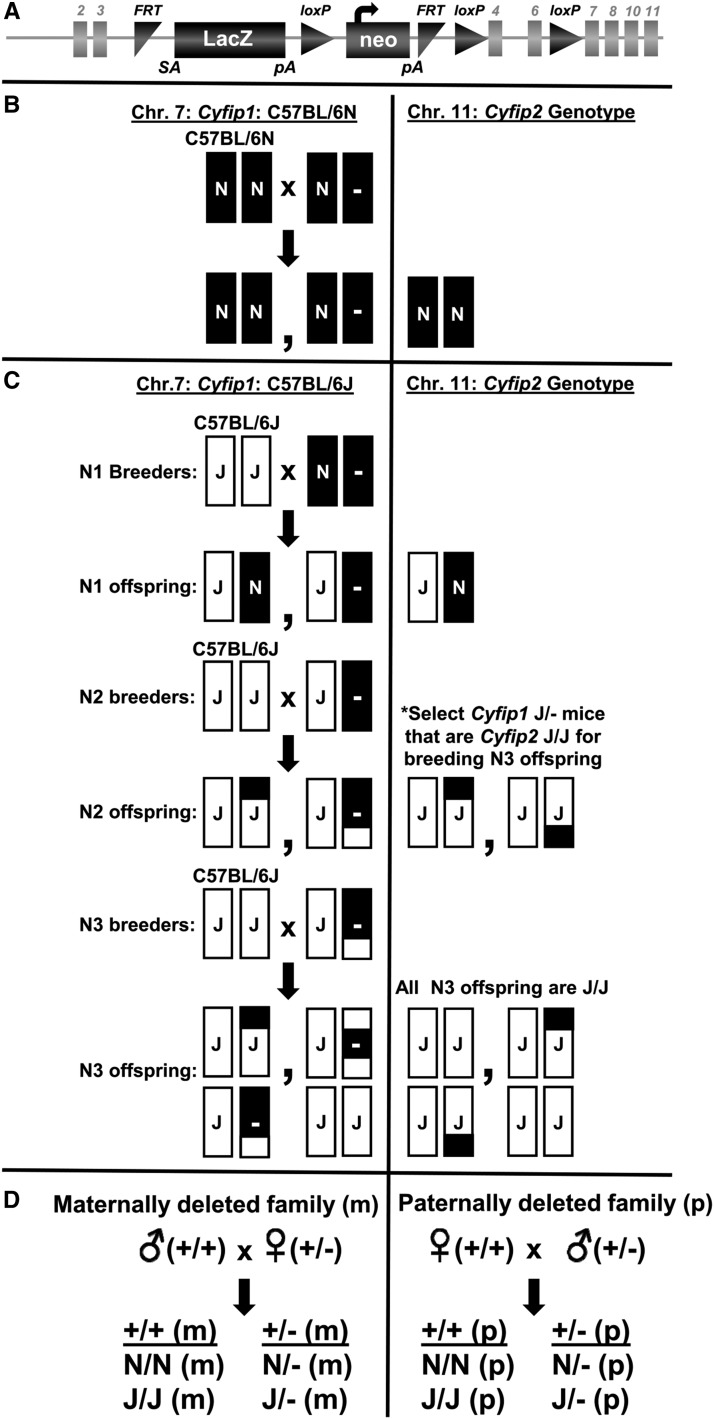
Generation of the *Cyfip1* knockout allele and breeding scheme for *Cyfip1* haploinsufficient mice on the *Cyfip1,2*^N/N^ and *Cyfip1,2*^J/J^ genetic backgrounds. (A): A schematic of the knockout first allele for KOMP generation of *Cyfip1*^N/-^ mice was obtained from the International Mouse Phenotyping Consortium (IMPC) website (http://www.mousephenotype.org/data/alleles/MGI:1338801/tm2a(EUCOMM)Wtsi). Mice containing floxed alleles flanking exons 4 through 6 were generated from embryonic stem cells on a C57BL/6N background by the International Knockout Mouse Consortium and were crossed to global Cre-expressing mice, yielding offspring heterozygous for constitutive deletions in exons 4 through 6. Mice heterozygous for the null deletion on a C57BL/6N background were re-derived using sperm obtained from The Jackson Laboratory. (B): Left panel: In the first study, we re-derived *Cyfip1*^N/-^ and propagated mice on an isogenic C57BL/6N background. Right panel: All mice were homozygous for the N allele (N/N) at *Cyfip2* which contains a missense mutation that we previously showed was associated with a marked enhancement of binge eating (BE), accounting for one-third of the genetic variance in parental strain BE (Kirkpatrick *et al.* 2017). We maintained this colony on an isogenic C57BL/6N background by breeding *Cyfip1*^N/-^ mice with C57BL/6NJ mice (black bars; N/N) ordered from The Jackson laboratory. (C): In the second study, we generated another colony on a mixed background. The primary goal was to monitor and replace the BE-associated N/N *Cyfip2* alleles with C57BL/6J (J/J) alleles via backcrossing *Cyfip1*^N/-^ mice to C57BL/6J (white bars; J/J) for three and four generations and assess the effect of *Cyfip1* deletion on BE on a mixed N3 and N4 background containing a fixed, BE-resistant, homozygous J/J genotype at *Cyfip2* (Kirkpatrick *et al.* 2017). Mixed-color bars illustrate hypothetical recombination events that accumulate through repeated backcrossing to C57BL/6J (white). (D): Schematic of the bidirectional, parent-of-origin crosses for generating wild-type (+/+) and heterozygous (+/−) offspring from either the paternally (p) deleted *Cyfip1* families or the maternally (m) deleted *Cyfip1* families. There are eight possible annotations, including four on the *Cyfip1*,2^N/N^ background and four on the *Cyfip1*,2^J/J^ background. N/N (m): Wild-type offspring from a maternally deleted family on a *Cyfip1*,2 N/N background; N/- (m): Heterozygous offspring from a maternally deleted family on a *Cyfip1*,2 N/N background; N/N (p): Wild-type offspring from a paternally deleted family on a *Cyfip1*,2 N/N background; N/- (p): Heterozygous offspring from paternally deleted family on a *Cyfip1*,2 N/N background; J/J (m): Wild-type offspring from a maternally deleted family on a *Cyfip1*,2 J/J background; J/- (m): Heterozygous offspring from a maternally deleted family on a *Cyfip1*,2 J/J background; J/J (p): Wild-type offspring from paternally deleted family on a *Cyfip1*,2 J/J background; J/- (p): Heterozygous offspring from a paternally deleted family on a *Cyfip1*,2 J/J background.

Sample sizes are listed in Supplementary Table 1. Figure 1 illustrates the breeding scheme for generating *Cyfip1*^+/−^ mice on two *Cyfip1,2* genetic backgrounds as well as the breeding scheme and annotations of the offspring derived from the bidirectional, parent-of-origin crosses. The full set of statistical results, including F statistics, p-values, and slope analyses are provided in the Figure Legends. Specific descriptions of each ANOVA model as well as p-values for main effects, interactions, and post-hoc group comparisons that are relevant to the conclusions are provided in the main text of the Results section below.

**Figure 2:**
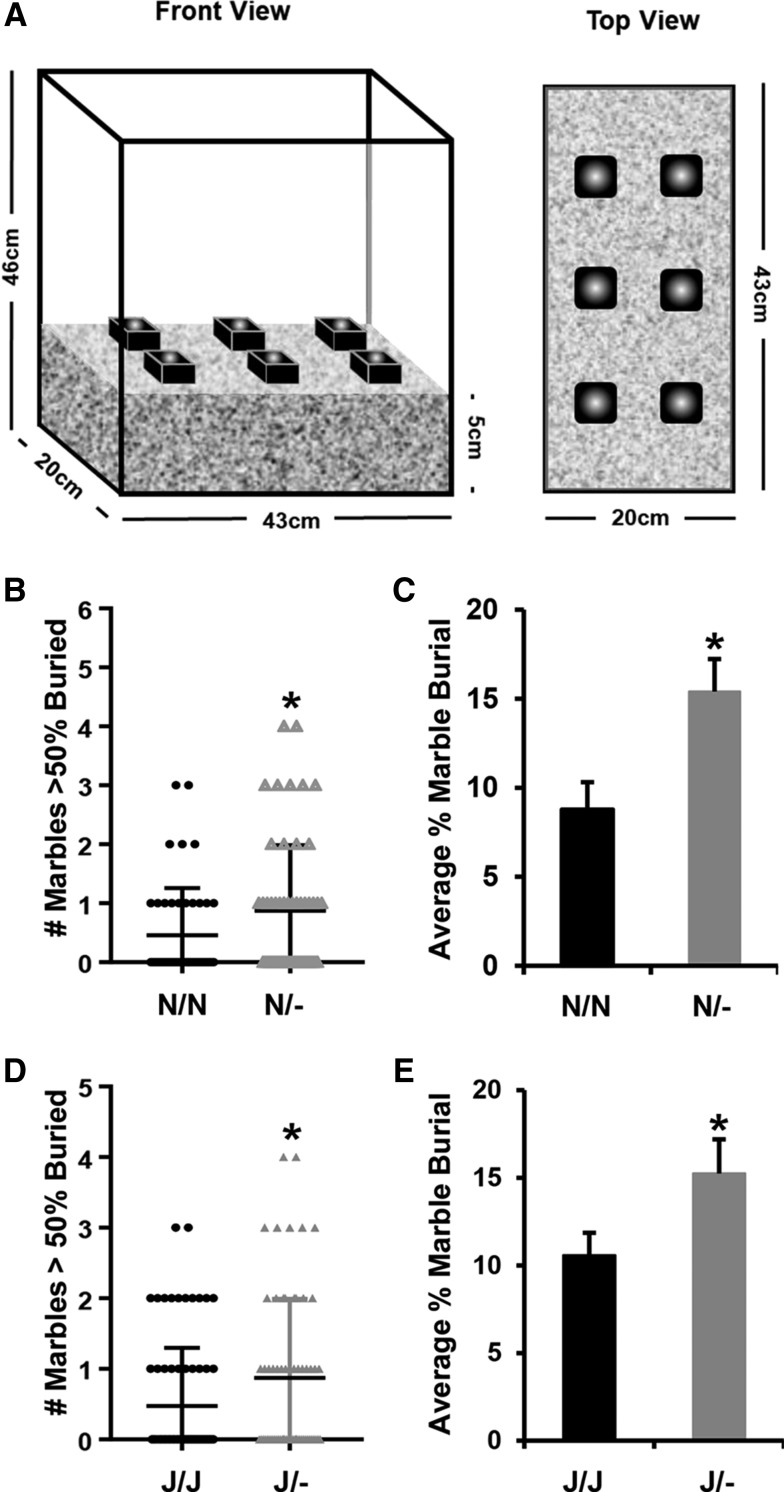
Increase in premorbid, OC-like marble burying in *Cyfip1*^N/-^ and *Cyfip1*^J/-^ mice. (A): Schematic of the marble burying apparatus. (B,C): *Cyfip1*^N/-^ mice buried more marbles with greater than 50% coverage than wild-type *Cyfip1*^N/N^ mice [B: U(102) = 1050; *P = 0.031; two-tailed], and had a greater average percent marble burial across the six marbles [C: effect of Genotype: F(1,96) = 7.1; *P = 0.009; no effect of PO and no Genotype x PO interaction; p’s > 0.05]. (D,E): Similarly, *Cyfip1*^J/-^ mice buried more marbles with greater than 50% coverage than *Cyfip1*^J/J^ mice [D: U(137) = 1884; *P = 0.019, two-tailed], and also had a greater average percent marble burial across the six marbles [E: Effect of Genotype: F(1,134) = 4.2; *P = 0.042; no effect of PO and no Genotype x PO interaction; p’s > 0.05]. Data are presented as the mean ± SEM.

In the marble burying test, *Cyfip1*^N/-^ mice on the *Cyfip1,2*^N/N^ genetic background showed a greater number of marbles that were at least 50% buried (Mann-Whitney: *P = 0.031). Furthermore, two-way ANOVA (Genotype, PO) identified a main effect of Genotype (*P = 0.009), indicating a greater average percentage of marbles buried than *Cyfip1*^N/N^ mice (Figure 2A-C). We replicated these results in *Cyfip1*^J/-^ on the *Cyfip1,2*^J/J^ background (Mann-Whitney: *P = 0.019; Effect of Genotype: *P* = *0.042, respectively; Figure 2D,E). If we collapse across genetic background and run either a Mann-Whitney *U*-test for number of marbles that were greater than 50% buried or an unpaired *t*-test for average percent marble burial across the six marbles in *Cyfip1*^+/−^ *vs. Cyfip1*^+/+^ mice and we employ a corrected p-value for statistical significance that accounts for the 14 phenotypes across all five behavioral assays within the compulsive-like battery (*P* < 0.05/14 = 0.0036), the result is still statistically significant for both phenotypes [U(235) = 5787; *P* = 0.0014; t(241) = 3.31; *P* = 0.0011 respectively]. Furthermore, the effect of Genotype in the ANOVA model of the collapsed data for average percent marble burial also survives the multiple correction procedure [F(1,238) = 10.9; *P* = 0.0011]. Finally, the increase in marble burying in *Cyfip1*^+/−^ mice cannot be explained by a genotypic difference in locomotor activity as there was no significant difference in distance traveled in *Cyfip1*^+/−^ *vs. Cyfip1*^+/+^ mice [collapsed across genetic backgrounds: t(241) = 0.79; *P* = 0.43].

**Figure 3:**
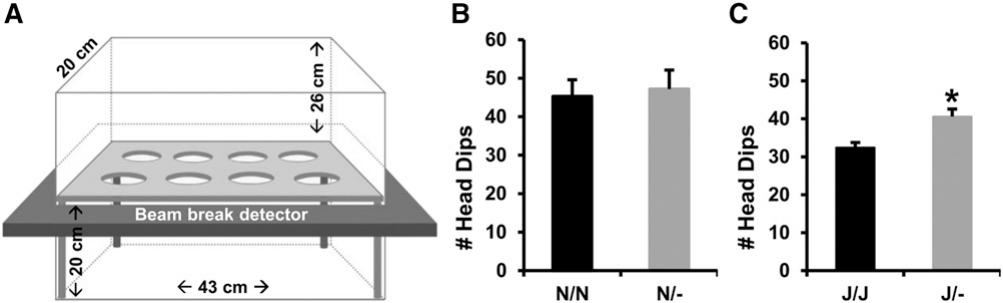
Hole board behavior in *Cyfip1*^N/-^ and *Cyfip1*^J/-^ mice. (A): Cartoon and dimensions of the hole board test. The holes (2 × 4) were 2.54 cm in diameter and were spaced center-to-center 10.16 cm apart. Head dips were detected via beam breaks (for additional details, see Supplementary Material). (B): In examining premorbid compulsive-like behavior in the hole board test, for mice on the *Cyfip1,2*^N/N^ background, *Cyfip1*^N/-^ mice did not differ from wild-type *Cyfip1*^N/N^ mice [t(102) = 0.29; *P* = 0.78]. (C): For mice on the *Cyfip1,2*^J/J^ background, *Cyfip1*^J/-^ mice showed a significantly greater number of head dips than *Cyfip1*^J/J^ mice [t(136) = 2.2; *P = 0.03]. Note that the significant increase in the number of head dips in *Cyfip1*^J/-^ mice does not survive the cut-off for significance if one corrects for all 14 phenotypes in the five-day behavioral battery (*P* < 0.0036; 12 phenotypes in Supplementary Table 2 plus two marble burying phenotypes in Fig. 2).

For the most part, *Cyfip1* deletion did not induce a statistically significant change in any other behaviors within the battery (Supplementary Table 2, *t*-tests: all ps > 0.05), including mist spray-induced grooming (p’s > 0.18; Supplementary Table 2). The only exception was that on the *Cyfip1,2*^J/J^ background, *Cyfip1*^J/-^ showed a greater number of total head dips in the hole board test compared to *Cyfip1*^J/J^ (*t*-test: *P* = 0.03; Figure 3), further supporting increased compulsive-like behaviors as a result of *Cyfip1* haploinsufficiency. The increase in head dips in *Cyfip1*^J/-^ mice cannot be explained by an overall increase in locomotor activity as an ANOVA model (Genotype and PO as factors) indicated that there was no significant effect of Genotype on distance traveled [F(1,134) = 3.44; *P* = 0.066] and despite the fact that *Cyfip1*^J/-^ mice showed a greater number of head dips than their *Cyfip1*^J/J^ counterparts, they actually tended to show less locomotor activity [8.49 +/− 0.30 m (SEM) *vs.* 9.23 +/− 0.27 m (SEM), respectively]. Note that the significant increase in head dips in *Cyfip1*^J/-^ mice is not statistically significant if one employs a Bonferroni-corrected p-value of 0.05/14 (*P* < 0.0036) to account for the 14 statistical tests across the five behaviors within the compulsive-/anxiety-like battery (12 phenotypes in Supplementary Table 2 plus the two marble burying phenotypes in Figure 2).

There was no main effect of PO or interaction with *Cyfip1* Genotype on marble burying or any other behaviors within the battery (data not shown). Furthermore, there were no significant genotypic differences in any of the other behaviors within the battery (Supplementary Table 2). To summarize, *Cyfip1* haploinsufficiency induced a selective increase in compulsive-like marble burying regardless of the genetic background or PO as well as an increase in compulsive-like head-dipping in the hole board test that was observed only on the *Cyfip1,2*^J/J^ genetic background. The lack of effect of *Cyfip1* haploinsufficiency on other OC-related behaviors such as mist spray-induced grooming could reflect differences in specific cell types and neural circuitry underlying complex repetitive action patterns for burying *vs.*, *e.g.*, grooming that are perturbed by *Cyfip1* haploinsufficiency (Kim *et al.* 2016).

### Effect of Cyfip1 haploinsufficiency on PF intake depends on Cyfip2 genetic background

**Figure 4:**
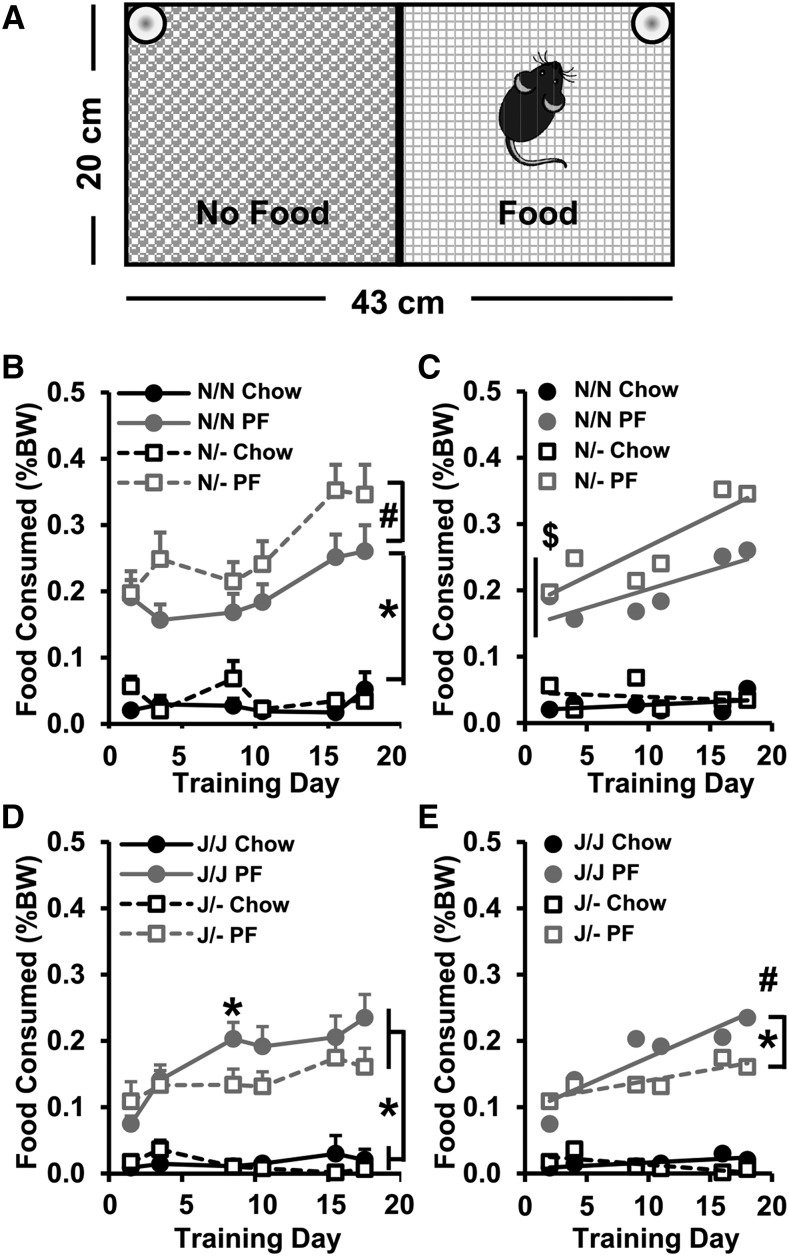
PF consumption in *Cyfip1*^N/-^ and *Cyfip1*^J/-^ mice. (A): The conditioned place preference (CPP) chamber that was used for food consumption training had a smooth-textured non-food-paired side (left) and a rough-textured food-paired side. (B): Both wild-type *Cyfip1*^N/N^ and *Cyfip1*^N/-^ mice trained with PF in the CPP chamber ate more food over time than Chow-trained mice [main effect of Treatment: F(1,932) = 274.7; *P < 2x10^−16^]. There was also a main effect of Day [F(5,932) = 16.1; *P* = 6.4 × 10-5], Sex [F(1,932) = 30.4; *P* = 4.5 × 10^−8^], a Treatment x Day interaction [F(5,932) = 7.9; *P* = 0.005], a Genotype x Treatment interaction [F(1,932) = 4.7; *P* = 0.03], and a Treatment x Sex interaction [F(1,932) = 22.3; *P* = 2.7 × 10^−6^]. *Cyfip1*^N/-^ mice consumed more PF overall than *Cyfip1*^N/N^ mice [summed intake across days: t(76) = 2.1; #*P* = 0.04; Fig.4B) but not Chow [summed intake across days: t(78) = 1.0; *P* = 0.32]. (C): Both PF-trained genotypes exhibited slopes that were significantly greater than zero (*Cyfip1*^N/N^: m = 0.009 ± 0.003, *P* = 0.024; *Cyfip1*^N/-^: m = 0.005 ± 0.002, *P* = 0.045, respectively, indicating escalation in PF intake over time. Moreover, PF-trained *Cyfip1*^N/-^ mice showed a significantly greater y-intercept than all three other groups ($p’s < 0.008 *vs.* each of the three groups), indicating consistently greater overall food consumption throughout the study. (D): When examining the same behaviors in *Cyfip1*^J/-^ *vs. Cyfip1*^J/J^ mice, there was a main effect of Treatment [F(1,978) = 191.1; *P = 2 × 10^−16^], indicating that PF-trained mice consumed more food over time. There was also a main effect of Genotype [F(1,978) = 5.4; *P* = 0.02], Day [F(5,978) = 4.0; *P* = 0.001], and a Treatment x Day interaction [F(5,978) = 2.6; *P* = 0.02] but in contrast to *Cyfip1* ^N/-^ mice, *Cyfip1*^J/-^ consumed *less* food than their wild-type *Cyfip1^J/J^* counterparts. There was a significant increase in PF intake on Day (D)9 in *Cyfip1*^J/J^ *vs. Cyfip1*^J/-^ mice [t(106) = 2.0; *P = 0.047]. Additionally, there was a main effect of Sex [F(1,978) = 15.7, *P* = 8.2 × 10^−5^], a Treatment x Sex interaction [F(1,978) = 6.0; *P* = 0.01], a Genotype x Sex interaction [F(1,978) = 4.9; *P* = 0.03], and most importantly, a Genotype x Treatment x Sex interaction [F(1,978) = 10.9; *P* = 0.001]. Follow-up sex-specific analyses for mice in the *Cyfip1*,2^J/J^ background are provided in Fig.5. (E): In examining escalation in food intake across days, only PF-trained *Cyfip1^J/J^* mice exhibited a slope significantly greater than zero [F(1,328) = 19.7; #*P* < 0.0001]. The slope value of *Cyfip1*^J/J^ mice was also greater than *Cyfip1*^J/-^ mice [F(1,644) = 4.0; *P = 0.046] and further supports reduced food intake in *Cyfip1*^J/-^ mice. Data are presented as mean ± SEM.

In testing the hypothesis that *Cyfip1* haploinsufficiency would increase PF intake in our intermittent, limited access BE and CPP paradigm (Figure 4A), Mixed-effects ANOVA (Factors: Genotype, Treatment, Sex; repeated measure: Day) indicated that PF-trained mice on the *Cyfip1,2^N/N^* background consumed significantly more food than Chow-trained mice (Figure 4B; effect of Treatment on intake [PF groups *vs.* Chow groups: *P < 2x10^−16^] – the Treatment effect was also reflected by slopes of escalation that were significantly greater than zero in the PF-trained groups (slopes *vs.* zero: *P* = 0.024-0.045; Figure 4C) but not in the Chow-trained groups. As predicted, *Cyfip1*^N/-^ mice consumed more PF than *Cyfip1*^N/N^ mice (Genotype x Treatment interaction; *P* = 0.03; *t*-test for summed PF intake: **#***P* = 0.04; Figure 4B) with no difference in Chow intake [*t*-test: *P* = 0.32]. There was also a main effect of Day [*P* = 6.4 × 10-5] and a Treatment x Day interaction [*P* = 0.005]. Finally, PF-trained *Cyfip1*^N/-^ mice showed a greater y-intercept than all three other groups ($p’s < 0.008 *vs.* each of the three groups; Figure 4C), indicating an initial higher level of consumption during early training days that persisted throughout the study.

In examining the effect of *Cyfip1* haploinsufficiency on food intake on the *Cyfip1,2*^J/J^ genetic background, as expected (Babbs *et al.* 2018; Goldberg *et al.* 2017; Kirkpatrick *et al.* 2017), there was less overall PF intake in mice on the *Cyfip1,2*^J/J^ background compared to the *Cyfip1,2*^N/N^ background [summed PF intake of mice on the *Cyfip1*,2^J/J^ *vs. Cyfip1*,2^N/N^ background: t(186) = 3.8; *P* = 0.001; not shown graphically but compare the two PF groups in Figure 4D *vs.* Figure 4B]. Furthermore, mixed-effects ANOVA of mice on the *Cyfip1*,2^J/J^ background (Factors: Genotype, Treatment, Sex; Repeated measure: Day) revealed that PF-trained mice showed greater intake than Chow-trained mice (effect of Treatment: *P = 2 × 10^−16^; Figure 4D). There was also a main effect of Genotype (*P* = 0.02), Day [*P* = 0.001) and a Treatment x Day interaction (*P* = 0.02) that were in part explained by a significant increase in PF intake on D9 in *Cyfip1*^J/J^ *vs. Cyfip1*^J/-^ mice [*P = 0.047; Figure 4D). Slope analysis identified a significant, BE-like slope in escalation of PF intake relative to zero in wildtype *Cyfip1*^J/J^ mice (**#***P* < 0.0001) that was significantly greater than the slope value of *Cyfip1*^J/-^ mice (*P = 0.046; Figure 4E).

Approximately one-half of the mice on the *Cyfip1*,2^J/J^ background had previously undergone prior training in the five-day behavioral battery which had a significant effect on PF intake. Specifically, in an ANOVA model of averaged food intake (collapsed across days) as the dependent measure and Battery, Genotype, and Treatment as factors, there was a significant effect of Battery [F(1,1018) = 18.0; *P* = 2.38 e-05] and a Battery x Treatment interaction [F(1,1018) = 14.0; *P* = 0.0002]. Subsequent ANOVA of PF intake alone identified PF treatment as the main source for the effect of Battery on food intake [Effect of Battery with PF-trained mice only: F(1,644) = 33.61; *P* = 1.06 × 10-8] that was explained by greater average PF intake across days in Battery-exposed mice (0.20 +/− 0.03% body weight consumed) compared to Battery-naïve mice (0.12 +/− 0.02% body weight consumed). Importantly, there was no significant Battery x Genotype interaction in either ANOVA model [F(1,1018) < 1; F(1,644) < 1, respectively], nor was there a significant Battery x Genotype x Treatment interaction [F(1,1018) <1]. Thus, prior training in the Battery increased PF intake and overall phenotypic variance without interacting with Genotype.

### PO- and sex-dependent effects of Cyfip1 haploinsufficiency on PF intake

We next investigated the effect of PO of *Cyfip1* deletion on food intake in the same data from Figure 4 in light of a recent study demonstrating a PO effect of *Cyfip1* deletion on emotional learning and synaptic transmission (Chung *et al.* 2015). We focused on PF intake rather than Chow intake based on the above results (Figure 4).

**Figure 5:**
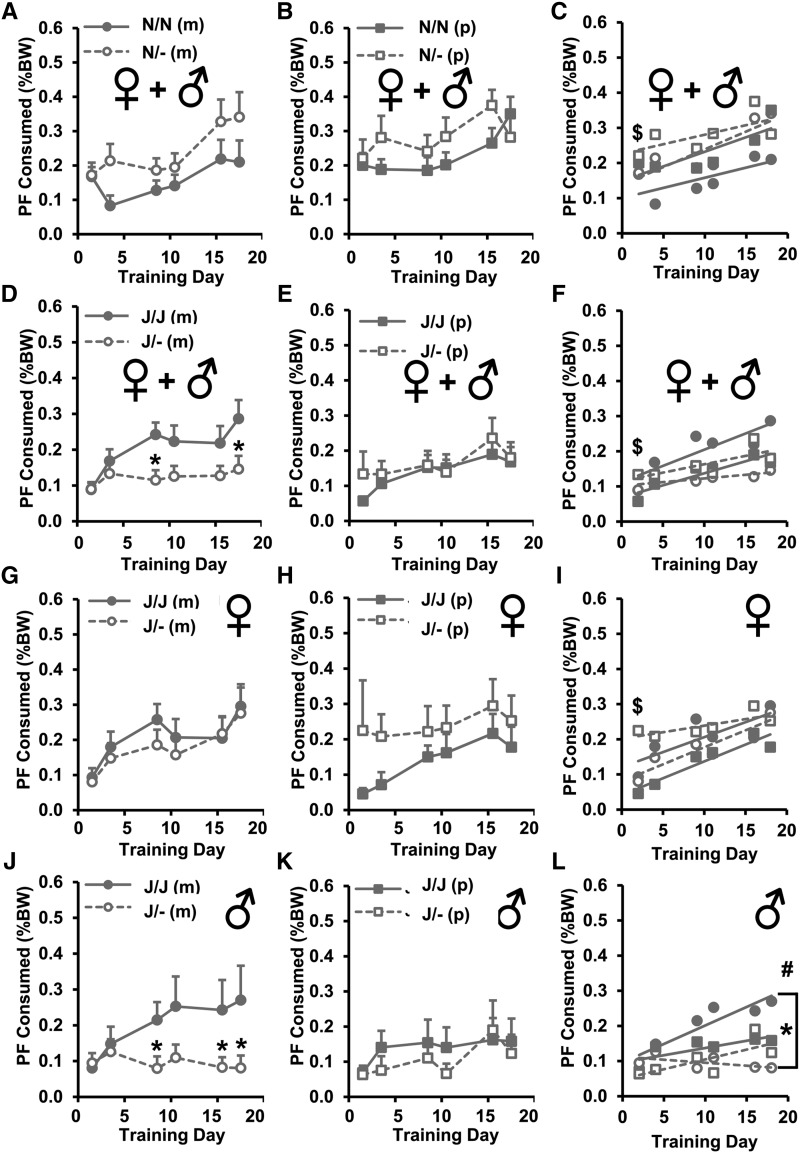
Effect of Parent-of-Origin (PO) and Sex on PF consumption in *Cyfip1*^N/-^ and *Cyfip1*^J/-^ mice. (A,B): For the *Cyfip1,2*^N/N^ background, there was an effect of Genotype [F(1,464) = 12.3; *P* = 0.0005], PO [F(1,464) = 9.0; *P* = 0.003], Sex [F(1,464) = 39.4; *P* = 8.0 × 10^−10^], and Day [F(1,464) = 20.8, *P* = 6.5 × 10^−6^]. *Cyfip1*^N/-^ mice consumed more PF than *Cyfip1*^N/N^ mice on Day (D)4 [A: t(29) = 2.1; *P = 0.046]. Females consumed more PF than males (not shown). (C): No differences were observed among the groups in the slopes of escalation in PF consumption [F(3,16) = 0.7 *P* = 0.56]; however, paternally-deleted *Cyfip1*^N/-^ mice (open squares) showed a greater y-intercept than either of the *Cyfip1*^N/N^ wild-type groups ($: both p’s < 0.02), indicating a greater overall consumption. Furthermore, *Cyfip1*^N/N^ mice derived from paternal deletion showed a greater y-intercept than *Cyfip1*^N/N^ mice derived from maternal deletion (*P* = 0.046). (D,E): For the *Cyfip1,2*^J/J^ background, there was an effect of Genotype [F(1,600) = 5.2; *P* = 0.02], Sex [F(1,600) = 14.1; *P* = 0.0002], Day [F(5,600) = 4.4; *P* = 0.0006], a Genotype x Sex interaction [F(1,600) = 10.8; *P* = 0.001], and a Genotype x PO interaction [F(1,600) = 9.3; *P* = 0.002] that reflected less PF consumption in maternally deleted *Cyfip1*^J/- (m)^ mice [D: t(59) = 2.9, 2.2; *P = 0.005, 0.03 *vs.* their *Cyfip1*^J/J^ counterparts on Day (D)9 and D18, respectively] but no genotypic differences between paternally deleted *Cyfip1*^J/- (p)^ mice *vs.* their *Cyfip1* ^J/J (p)^ counterparts (E). (F): Both maternal and paternal wild-type *Cyfip1*^J/J^ groups showed significant slope in escalation of intake [*Cyfip1*^J/J (m)^: F(1,184) = 10.9; *P* = 0.001; *Cyfip1* ^J/J (p)^: F(1,142) = 8.4; *P* = 0.005; closed symbols]. In contrast, neither mutant *Cyfip1*^J/-^ group showed a significant slope from zero (both ps > 0.15; open symbols). Moreover, paternally deleted *Cyfip1*^J/- (p)^ mice (open squares) had a greater y-intercept than all three other groups ($ all p’s < 0.0004). (G-I): To understand the source of the above interactions, we next separated PO effects of *Cyfip1^J/^*^-^ by Sex. (G-H): In females, there was an effect of Day [F(5,288) = 3.6; *P* = 0.004] and a Genotype x PO interaction [F(1,288) = 7.1; *P* = 0.008]. (I): Both *Cyfip1^J/J^* wild-type female groups showed a significant slope in escalation (I; both ps < 0.02 *vs.* zero) as well as maternally-deleted Cyfip1^J/- (m)^ females (*P* = 0.002 *vs.* zero). Paternally deleted *Cyfip1*^J/- (p)^ mice did not show a significant non-zero slope (*P* = 0.5) but showed the greatest y-intercept compared to all three groups ($*P* < 0.0002), indicating an initially higher, stable PF consumption across time. (J-L): For males, there was an effect of Genotype [F(1,312) = 13.6; *P* = 0.0003] and a trending Genotype x PO interaction [F(1,312) = 3.3; *P* = 0.07]. Maternally deleted *Cyfip1*^J/- (m)^ males showed significantly less PF intake than their wild-type *Cyfip1*^J/J (m)^ male counterparts (J: *P < 0.03, 0.04, and 0.04 *vs. Cyfip1*^J/J^ on D9, D16, and D18, respectively). In contrast, there was no genotypic difference in paternally-deleted *Cyfip1*^J/- (p)^ males *vs.* their wild-type *Cyfip1*^J/J (p)^ male counterparts (panel K). For slope analysis, only the wild-type *Cyfip1* ^J/J (m)^ males showed a significant slope in escalation of consumption (#*P* = 0.03 *vs.* zero) that was also significantly greater than their mutant *Cyfip1*^J/- (m)^ male counterparts (*P = 0.01; L). Data are presented as mean ± SEM.

In examining the effect of PO on PF intake on the *Cyfip1,2*^N/N^ background, mixed-effects ANOVA (Factors: Genotype, PO, Sex; repeated measure: Day) revealed a main effect of PO (*P* = 0.003) and Day (*P* = 6.5 × 10^−6^; Figure 5A,B). *Cyfip1*^N/- (p)^ mice showed a greater y-intercept compared to their wild-type *Cyfip1*^N/N (p)^ counterparts and the other wild-type *Cyfip1*^N/N (m)^ group ($p’s <0.02; Figure 5C). Furthermore, wild-type *Cyfip1*^N/N (p)^ offspring from paternally deleted families showed a greater y-intercept than wild-type *Cyfip1*^N/N (m)^ offspring from maternally deleted families (*P* = 0.046; Figure 5C).

For the *Cyfip1,2*^J/J^ background, mixed effects ANOVA (Factors: Genotype, PO, Sex; repeated measure: Day) revealed a Genotype x PO interaction on PF intake (*P* = 0.002) and an effect of Day (*P* = 0.0006). Maternal *Cyfip1* deletion [*Cyfip1*^J/- (m)^] accounted for the reduced intake in *Cyfip1*^J/-^ mice that we reported in Figure 4D whereby *Cyfip1* ^J/- (m)^ mice showed decreased PF intake compared to *Cyfip1*^J/J (m)^ mice on D9 and D18 (*t*-tests: *P* = 0.005, 0.03; Figure 5D). There was no genotypic difference in PF intake in offspring derived from paternal *Cyfip1* deletion (Figure 5E). Maternal *Cyfip1* deletion induced a significant slope in escalation of PF intake from zero in *Cyfip1*^J/J (m)^ mice (*P* = 0.001) but not *Cyfip1*^J/- (m)^ mice (Figure 5F).

For the *Cyfip1,2*^J/J^ background, we also observed a Genotype x Sex interaction in PF intake (*P* = 0.001). To identify the source of this interaction we broke down the maternal and paternal data separately into females and males. Paternally deleted female *Cyfip1*^J/- (p)^ mice initially showed *enhanced* PF intake relative to their female wild-type *Cyfip1*^J/J (p)^ counterparts as hinted by a trending increase in D4 PF intake (t(20) = 2.0; *P* = 0.06; Figure 5H) that was further supported by a statistically significant increase in y-intercept ($) relative to all three other groups (*P* = 0.0002 *vs. Cyfip1*^J/J (m)^ ; *P* = 0.0002 *vs. Cyfip1*^J/- (m)^; *P* < 0.0001 *vs. Cyfip1*^J/J (p)^; Figure 5I). In contrast, maternally-deleted male *Cyfip1*^J/- (m)^ mice showed a marked *decrease* in PF intake relative to male *Cyfip1*^J/J (m)^ mice on D9, D16, and D18 (*t*-tests: *P* = 0.03, 0.04, 0.04) and no significant increase in slope of escalation *vs.* zero (Figure 5J-L). In contrast, male wild-type *Cyfip1* ^J/J (m)^ mice showed a significant slope of escalation in PF intake (*Cyfip1*^J/J (m)^ slope *vs.* zero: *P* = 0.03) that was significantly greater than their male *Cyfip1* ^J/- (m)^ counterparts (*vs.* J/- (m): *P* = 0.01) but not greater than paternally deleted male wild-type *Cyfip1* ^J/J (p)^ (*P* = 0.29) or mutant *Cyfip1*^J/- (p)^ mice (*P* = 0.39; Figure 5L).

To summarize, we observed opposite effects of *Cyfip1* deletion on PF intake that depended on *Cyfip1,2* genetic background, PO, and Sex. Despite changes in PF intake across Genotype and PO, differences in body weight cannot fully account or the complex interactive effects of Genotype, PO on PF intake (Supplementary Figure 1). I.e., homeostatic mechanisms are unlikely to account for group differences in PF intake.

### Conditioned food reward in Cyfip1^N/-^ and Cyfip1^J/-^ haploinsufficient mice

In examining food CPP via the change in preference for the food-paired side (s) between D1 and D22 of training on the *Cyfip1,2*^N/N^ genetic background, two-way ANOVA (Factors: Genotype, Treatment) revealed no main effect of *Cyfip1* Genotype, Treatment, or interaction in mice from either genetic background (p’s > 0.13; Supplementary Figure 2A,B). However, when considering PF treatment alone (as we did for a subset of the above analyses involving PF intake), there was increased PF-CPP in *Cyfip1*^N/-^ *vs. Cyfip1*^N/N^ mice that was consistent with increased PF intake (*t*-test: *P* = 0.03; Supplementary Figure 2A). For *Cyfip1*^J/-^ mice on the *Cyfip1,2*^J/J^ background, there was no genotypic difference in PF-CPP (Supplementary Figure 2B). In considering the effect of PO on PF-CPP, there was no effect of Genotype, PO, or interaction for either *Cyfip1,2* genetic background (data not shown).

### Compulsive-like eating in the light/dark conflict test in Cyfip1^N/-^ and Cyfip1^J/-^ haploinsufficient mice

**Figure 6:**
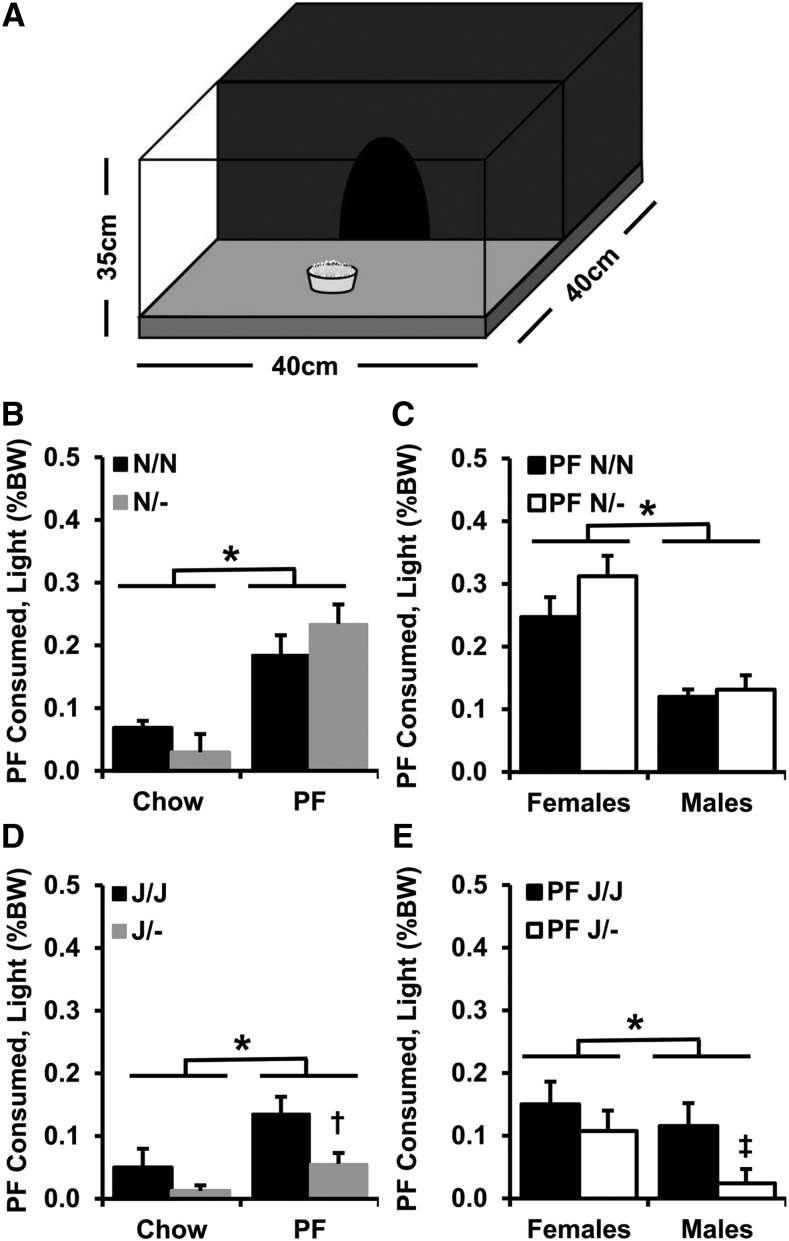
Compulsive-like PF intake in the light/dark conflict test in *Cyfip1*^N/-^ and *Cyfip1*^J/-^ mice. (A): A cartoon of the apparatus for the light/dark conflict test of compulsive-like PF consumption is shown. (B): For the *Cyfip1,2*^N/N^ background, there was a main effect of Training Treatment [F(1,148) = 31.1; *P = 1 × 10^−7^], indicating increased PF intake. However, there was no effect of Genotype [F(1,148) = 0.1; *P* = 0.7] or Genotype x Training Treatment interaction in consumption [F(1,148) = 2.3; *P* = 0.13]. (C): In examining PF-trained mice alone, females showed increased intake [effect of Sex: F(1,72) = 13.6; *P = 0.0004]; however, there was no effect of Genotype [F(1,72) = 1.2; *P* = 0.3] or Genotype x Sex interaction [F(1,72) = 0.7; *P* = 0.4]. (D): For mice on the *Cyfip1,2*^J/J^ genetic background, there was a main effect of Training Treatment [F(1,163) = 8.5; *P = 0.004], indicating greater PF intake in PF-trained mice. There was also a main effect of Genotype [F(1,163) = 9.8; *P* = 0.002] that was explained primarily by less PF intake in *Cyfip1^J/^*^-^ mice *vs. Cyfip1^J/J^* mice [t(92) = 2.8; †*P* = 0.007]. (E): In considering only PF-trained mice, females trended toward greater overall intake [effect of Sex: F(1,100) = 3.9; *P = 0.05] and *Cyfip1*^J/-^ mice showed overall less intake than *Cyfip1^J/J^* mice [effect of Genotype: F(1,100) = 7.6; *P* = 0.007] that was explained primarily by males [‡: t(54) = 2.7; *P* = 0.01]. Data are presented as the mean ± SEM.

We next examined post-training compulsive-like PF intake using the light/dark conflict test (Figure 6A) (Babbs *et al.* 2018; Kirkpatrick *et al.* 2017). Separate, three-way ANOVA (Factors: Genotype, Treatment, Sex) for each of the two genetic backgrounds (*Cyfip1*,2^N//N^, *Cyfip1*,2^J/J^) revealed that PF-trained mice showed greater PF intake in the light/dark arena than Chow-trained mice (effect of Treatment: *P = 1 × 10^−7^ and 0.004, respectively; Figure 6B,D). Furthermore, overall, females showed greater PF intake than males on both genetic backgrounds (effect of Sex: *P* = 0.0004 and 0.05, respectively; Figure 6C,E). For mice on the *Cyfip1,2*^N/N^ background, there was no genotypic difference in PF consumption in the light/dark arena (Figure 6B,C), regardless of PO (Supplementary Figure 3A-B). For mice on the *Cyfip1,2*^J/J^ background, *Cyfip1*^J/-^ mice showed reduced PF consumption (effect of Genotype: *P* = 0.002; *t*-test: **†***P* = 0.007; Figure 6D) that was driven primarily by the males (*t*-test: **‡***P* = 0.01; Figure 6E).

### Reduced transcription of Cyfip1 but not Cyfip2 or Magel2 in the hypothalamus of Cyfip1^+/−^ mice

We hypothesized that the PO- and genetic background-dependent effects of *Cyfip1* deletion on PF intake could involve differences in hypothalamic gene transcription of *Cyfip1* and other genes, including *Cyfip2* and *Magel2*. Supplementary Table 3 lists the qPCR results as a function of both *Cyfip1* haploinsufficiency and PO. For the *Cyfip1,2*^N/N^ background, there was a reduction in *Cyfip1* transcription in *Cyfip1^N^*^/-^ mice following either maternal (*t*-test: *P* = 0.04) or paternal *Cyfip1* deletion (*t*-test: *P* = 0.04; Supplementary Table 3A) as these two groups did not differ from one another [t(14) < 1]. In contrast, when the effect of *Cyfip1* haploinsufficiency on *Cyfip1* transcription was assessed on the *Cyfip1,2*^J/J^ background, maternally deleted *Cyfip1* ^J/- (m)^ mice showed a significant decrease in Cyfip1 transcription relative to wild-type *Cyfip1*^J/J^ (*t*-test: *P = 0.02) and relative to paternally deleted *Cyfip1*^J/- (p)^ mice (*t*-test: *P* = 0.0061; Supplementary Table 3B). There were no genotypic differences in hypothalamic transcription of *Cyfip2* or *Magel2* (Supplementary Table 3).

### Reduced CYFIP1 protein expression in Cyfip1^+/−^ mice depends on PO

**Figure 7:**
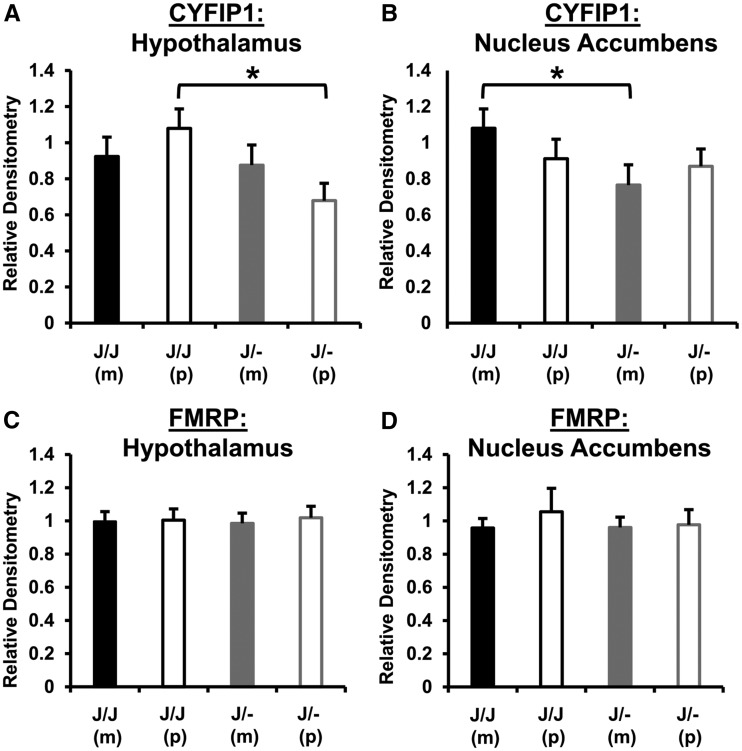
CYFIP1 and FMRP protein expression in the hypothalamus and nucleus accumbens. For CYFIP1 and FMRP, there was no effect of Sex (ps > 0.1) or interaction of Sex with other factors (ps > 0.30). There was also no effect of prior Treatment or interaction of Treatment with other factors (ps > 0.10). Therefore, we collapsed across Sex and Treatment. The majority of samples used in CYFIP1 analysis were from PF-trained mice (60 to 61 PF-trained samples per brain region; 16 to 18 Chow-trained samples per brain region) and trending results for the same statistics reported below were observed when analyzing only the PF-trained samples (p’s = 0.065-0.15). Similarly, the majority of samples used for FMRP analysis were from PF-trained mice for hypothalamus (31 to 48 PF-trained samples per brain region; 0 to 8 Chow-trained samples per brain region) and for the nucleus accumbens, all samples were PF-trained (0 Chow-trained). The same null results were observed in the hypothalamus for the effect of Genotype (*P* = 0.43), PO (*P* = 0.86), and interaction (*P* = 0.26). (A): In examining CYFIP1 protein levels in the hypothalamus via immunoblotting, there was a main effect of Genotype [F(1,73) = 7.1; *P* = 0.009] and a Genotype x PO interaction [F(1,73) = 4.; *P* = 0.039]. Paternally deleted *Cyfip1*^J/- (p)^ mice showed lower protein expression than their wild-type *Cyfip1*^J/J (p)^ counterparts [t(36) = 3.0; *P = 0.0055]. (B): In examining CYFIP1 protein levels in the nucleus accumbens, the effect of Genotype was not significant [F(1,72) = 2.9; *P* = 0.09]. Maternally deleted *Cyfip1*^J/-(m)^ mice showed a lower level of immunostaining for CYFIP1 protein than their wild-type *Cyfip1*^J/J (m)^ littermates [t(37) = 2.0; *P = 0.049]. (C): In examining FMRP levels in the hypothalamus, there was no effect of Genotype, PO, or Genotype x PO interaction [F(1,71) < 1]. (D): In examining FMRP levels in the nucleus accumbens, all samples were from PF-trained mice. Densitometry analysis revealed no effect of Sex or interaction of Sex with any other factors [F(1,23) <`1]. Analysis of FMRP immunoblots from nucleus accumbens revealed no effect of Genotype, PO, or Genotype x PO interaction [F(1,27) < 1].

Because preliminary evidence indicated possible effects of PO on Cyfip1 expression at the mRNA level in *Cyfip1*^J/-^ mice on a *Cyfip2*^J/J^ background (Supplementary Table 3), we next investigated CYFIP1 expression at the protein level on the same genetic background. We examined the hypothalamus as well as nucleus accumbens. In the hypothalamus, paternally deleted *Cyfip1*^J/- (p)^ mice showed a significant decrease in CYFIP1 protein relative to their respective *Cyfip1* ^J/J (p)^ wild-type mice as measured via immunoblot (*t*-test: *P* = 0.0055; Figure 7A). In the nucleus accumbens, maternally-deleted *Cyfip1*^J/- (m)^ mice showed a significant decrease in CYFIP1 protein expression relative to their respective *Cyfip1* ^J/J (m)^ wild-type mice (*t*-test: *P* = 0.049; Figure 7B; Supplementary Figure 4). Thus, either maternal or paternal *Cyfip1*^+/−^ can result in an enhanced reduction in CYFIP1 protein expression, depending on the brain region.

### No effect of CYFIP1 haploinsufficiency on FMRP protein expression

*Fmr1* could potentially explain Sex- and PO-specific effects of *Cyfip1*^+/−^ on PF intake because 1) it is located on the X chromosome; 2) its protein product FMRP interacts with CYFIP1 (Abekhoukh and Bardoni. 2014); and 3) similar maternal effects of *Fmr1* haploinsufficiency on behavior in wild-type male mice have been reported on locomotor activity in male mice (Gleason *et al.* 2011; Zupan and Toth. 2008). We examined locomotor activity in offspring derived from *Cyfip1*^J/-(m)^ and *Cyfip1*^J/- (p)^ during initial preference assessment on D1 in the CPP apparatus and again, observed a selective effect of *Cyfip1* haploinsufficiency on behavior in maternally deleted male *Cyfip1*^J/- (m^) mice that showed increased locomotor activity relative to their wild-type *Cyfip1*^J/J (m)^ counterparts (Supplementary Figure 5). Therefore, we examined FMRP expression in *Cyfip1*^J/-^ *vs. Cyfip1*^J/J^ mice but found no effect of *Cyfip1* Genotype or PO on FMRP protein levels in the hypothalamus or nucleus accumbens via immunoblot (Figure 7C,D; Supplementary Figure 6). These null results do not support the hypothesis that FMRP is involved in the downstream mechanisms underlying Sex- and PO-specific effects of *Cyfip1*^J/-^ haploinsufficiency on behavior.

## Discussion

*Cyfip1* haploinsufficiency increased OC-like behavior on two different *Cyfip2* genetic backgrounds (Figures 1-3) and altered PF consumption and *Cyfip1* gene expression at the RNA and protein level, depending on genetic background (*Cyfip1,2*), PO, and Sex (Figures 4,5,7; Supplementary Table 3). These findings identify a significant contribution of *CYFIP1* haploinsufficiency to OC-like behaviors and PF intake that could have relevance for neurodevelopmental disorders (*e.g.*, Type I PWS and FXS) and for neuropsychiatric disorders (*e.g.*, OCD, eating disorders). Sex differences in PWS hyperphagia have not been widely reported (Irizarry *et al.* 2015). Our findings suggest the possibility of sex differences in PWS hyperphagia, specifically with Type I PWS, which could have implications for developing sex-specific pharmacotherapeutic treatments.

The relatively selective increase in OC-like but not anxiety-like behavior following *Cyfip1* deletion (Figure 2; Supplementary Table 2) is consistent with a lack of genetic correlation between marble burying and anxiety and supports marble burying as a repetitive, perseverative-like behavior (Thomas *et al.* 2009). Nevertheless, there is likely an anxiety-like component to marble burying (Albelda and Joel. 2012) as there is with OC behaviors in humans. For the *Cyfip1,2*^J/J^ genetic background, the increase in head-dipping behavior in the hole board task in *Cyfip1*^J/-^ mice (Figure 3) further supports an increase in OC-like/anxiety-like behaviors (Takeda *et al.* 1998) following *Cyfip1* haploinsufficiency, although it should be noted that in contrast to the marble burying behavior (see Results), the p-value for statistical significance for head dipping in the hole board does not survive correction for the 14 statistical tests across the battery of five behavioral assays (*P* < 0.0036).

*Cyfip1*^+/−^ mice showed an increase in marble burying which has been shown to predict BE (Freund *et al.* 2015; Satta *et al.* 2016). However, in our studies, there was no clear relationship between OC-like behaviors and PF intake because *Cyfip1* haploinsufficiency increased marble burying on both *Cyfip1*,2 genetic backgrounds (Figure 2) yet had opposite effects on PF consumption, depending on the background (Figures 4-5). These results effectively dissociate increased OC-like behavior from increased PF intake following *Cyfip1* haploinsufficiency. This dissociation is also evident in patients with PWS who show an increase in OC behaviors that is unrelated to food and is exacerbated in Type I PWS (with *CYFIP1* deletion) (Bittel *et al.* 2006; Butler *et al.* 2004; Doornbos *et al.* 2009; Milner *et al.* 2005; Zarcone *et al.* 2007). Thus, *CYFIP1* deletion could increase the severity of OC symptoms in Type I PWS without modulating eating behavior. Furthermore, multiple types of *CYFIP1* variants (structural, coding, intronic, upstream, intergenic) could act more broadly within the general population to associate with OC symptoms (Figures 2-3) or eating behavior in a manner that depends on genetic background (Figures 4-5).

The selective modulation of sweetened PF intake as evidenced during training (Figure 4) and during assessment of compulsive-like eating (Figure 6) combined with the selective demonstration of conditioned reward for sweetened PF (Supplementary Figure 2A) are observations that are consistent with increased preference for sweetened PF as a consequence of *Cyfip1* haploinsufficiency and are consistent with a role of *Cyfip* genes in modulating the hedonic aspects of food intake. In support, the *Cyfip2*^N/N^ S968F missense mutation in the closely related *Cyfip2* gene was associated with both cocaine neurobehavioral sensitivity and plasticity (Kumar *et al.* 2013) and increased compulsive-like BE (Kirkpatrick *et al.* 2017). Furthermore, differences in in *Cyfip2* mRNA expression genetically correlate with differences in cocaine self-administration in the BXD recombinant inbred strain panel (Dickson *et al.* 2015). We observed PO-dependent decreases in CYFIP1 protein in both the hypothalamus and nucleus accumbens (Figure 7), a brain region critical for the hedonic aspects of palatable food intake (Lutter and Nestler. 2009). Finally, previous transcriptome analysis of the striatum from *Cyfip2*^N/-^ *vs. Cyfip2*^N/N^ genotypes identified “morphine addiction” and “cocaine addiction” as two of the top five KEGG enrichment terms (Kirkpatrick *et al.* 2017). Together, these findings indicate that both *Cyfip1* and *Cyfip2* could alter the rewarding/hedonic response to PF consumption to affect food intake.

In contrast to our prediction, *Cyfip1*^J/-^ mice on the BE-resistant *Cyfip1,2*^J/J^ background did not show an escalation of PF intake (Figure 4D,E). Instead, we observed what appeared to be a decrease in PF intake in *Cyfip1*^J/-^ mice that was explained by the surprising *induction* of a BE phenotype (escalated PF intake) in wild-type mice on a *Cyfip1,2*^J/J^ background (Figure 4E). This observation was puzzling, given that we have repeatedly shown that mice (especially males) on a mixed F2 background with a homozygous *Cyfip2*^J/J^ genotype or on an isogenic C57BL/6J background do not show BE (Babbs *et al.* 2018; Goldberg *et al.* 2017; Kirkpatrick *et al.* 2017). Closer inspection revealed that the induction of BE in genetically unaffected wild-type mice was completely accounted for by wild-type male offspring derived from maternal *Cyfip1* deletion (*Cyfip1*^J/J (m)^; Figure 5J,L). Interestingly, although we failed to provide evidence for an association of FMRP expression with behavior (Figure 7C,D; Supplementary Figure 6), a similar pattern of results was observed with maternal haploinsufficiency of *Fmr1* (coding for FMRP) whereby genetically unaffected wild-type males demonstrated constitutive locomotor hyperactivity (Zupan and Toth. 2008) relative to wild-type males derived from wild-type dams. Thus, while our results do not support a link between *Cyfip1* haploinsufficiency, FMRP, and behavior, in the context of the prior *Fmr1* literature, they illustrate the importance of both Sex and PO as a biological variables when investigating the phenotypic effects of gene haploinsufficiency.

Genetic interactions with the social environment can contribute significantly to behavioral variance (Baud *et al.* 2017). Maternal *vs.* paternal *Cyfip1* deletion could affect social interactions with the dam and sire or with the maternal/paternal care of the pups. As an example, both genetically affected and unaffected male offspring derived from maternal *Fmr1* haploinsufficiency showed increased social approach behaviors toward conspecific strangers and neurobiological evidence supporting social aversion (Zupan *et al.* 2016). In addition to the maternal effects of gene deletion on neurobehavioral phenotypes of genetically unaffected offspring (Gleason *et al.* 2011), males can demonstrate paternal pup retrieval (Liu *et al.* 2013) and thus, paternal *Cyfip1* deletion could also affect sire-pup contact and behavior in the offspring. For example, selective effects of paternal deletion of neuregulin 1 on multiple behaviors of genetically affected male offspring have also been reported, including decreased fear learning and increased social interactions (Shang *et al.* 2017). Given the association between *CYFIP1* deletion and social deficits in neurodevelopmental disorders (Abekhoukh and Bardoni. 2014), *Cyfip1*^+/−^ in the dam or sire could affect the social dynamics in the offspring in a PO-dependent, genotype (offspring)-dependent, and sex-dependent manner, leading to long-term neurobehavioral effects. One hypothesis is that wild-type *Cyfip1*^J/J^ males are particularly susceptible to social influences of maternal-pup and/or pup-pup interactions in the maternally-deleted *Cyfip1*^J/-^ environment (whereas the *Cyfip1*^J/-^ mice are resistant), ultimately explaining the selective induction of escalated PF intake.

What is the mechanism underlying sex-dependent, PO-specific effects of *Cyfip1* deletion on behavior and gene expression? There is no published evidence that *Cyfip1* is imprinted and while our analysis of *Cyfip1* transcript and protein expression indicate PO-dependent effects as previously reported (Chung *et al.* 2015), the direction was not always consistent with maternal imprinting and was dependent on the particular brain region (Supplementary Table 3; Figure 7). A recent study of nearly 100 phenotypes showed that most complex traits exhibit PO effects and that non-imprinted KO alleles (*e.g.*, *Cyfip1*) can induce extensive PO effects by interacting in *trans* with imprinted loci throughout the genome to affect gene networks (Mott *et al.* 2014). If a *trans*-acting genomic mechanism underlies the effects of *Cyfip1* haploinsufficiency on behavior, the *trans*-acting factor(s) must be faithfully co-inherited with the maternal or paternal deletion to explain the specific PO effects. One such mechanism could involve inheritance of sex-dependent gene expression patterns originating from sex chromosomes that interact with *Cyfip1* deletion to affect neurobehavioral phenotypes. *Fmr1* is located on the X chromosome and codes for FMRP, an RNA-binding protein that interacts with CYFIP and regulates mRNA translation (Abekhoukh and Bardoni. 2014). However, despite observing *Fmr1*-like PO effects of maternal *Cyfip1* haploinsufficiency on initial locomotor activity prior to BE training (Supplementary Figure 5), we did not observe any evidence for a relationship between FMRP protein expression and the complex interactive effects of *Cyfip1* haploinsufficiency on PF intake (Figure 7C, D), nor did we observe any difference in FMRP expression between females and males [t(73)=1.68; *P* = 0.10]. These null results are consistent with *FMR1* undergoing X-inactivation (Kirchgessner *et al.* 1995) and fail to support a mechanistic role for FMRP, although we should note that differential FMRP expression could still be involved at some stage of neurodevelopment in the underlying mechanisms. Residual heterozygosity of B6NJ alleles on the X chromosome could also affect the expression of X-linked genes that act as modifiers of *Cyfip1* transcription or that skew X-inactivation and account for the background-dependent PO effects of *Cyfip1* deletion on behavior. The use of the four core genotypes model (XX, XY, XX-male, XY-female) could test the involvement of sex chromosomes in PO- and Sex-dependent effects of *Cyfip1* deletion on PF intake (Arnold and Chen. 2009). Notably, a recent study using this genetic model identified a contribution of sex chromosomes to operant reinforcement for PF (Seu *et al.* 2014).

Our preclinical findings provide evidence that reduced CYFIP1 expression could contribute to OC behaviors and disordered eating (Chang *et al.* 2019). Future genomic studies of brain regions, cell types, and neurodevelopmental time points could inform molecular mechanisms of eating behaviors on different genetic backgrounds and the potential interaction of *Cyfip1* deletion with gene expression on sex chromosomes.
